# Retraction: PDZ-containing 1 acts as a suppressor of pancreatic cancer by regulating PTEN phosphorylation

**DOI:** 10.18632/oncotarget.25033

**Published:** 2018-05-04

**Authors:** Qiang Ma, Xiuxiu Wu, Jing Wu, Huanwen Wu, Ying Xiao, Lili Wang, Zhiyong Liang, Tonghua Liu

**Affiliations:** ^1^ Department of Pathology, Peking Union Medical College Hospital, Chinese Academy of Medical Sciences and Peking Union Medical College, Beijing 100730, P. R. China; ^2^ Department of Respiratory Medicine, Peking Union Medical College Hospital, Chinese Academy of Medical Sciences and Peking Union Medical College, Beijing 100730, P. R. China; ^3^ Department of Medical Imaging, Beijing Huairou Hospital, University of Chinese Academy of Science, Beijing, 101400, P.R. China

Corresponding author Tonghua Liu requested a retraction of this paper, and an institutional committee at Peking Union Medical College Hospital confirmed this decision.

The authors' explanation is as follows:

Due to the authors' negligence during the preparation of this manuscript for submission, the reported data contain a number of errors. For example, in Figure 4B and 4C, the authors only report detecting the level of p-PTEN expression. Because the level of PTEN expression was neglected, those results are flawed. In Figure 5C, the time marks and the ordinate are incorrect. In Figure 6A, the legend does not appropriately reflect the figure. And in Figure 7D, the statistical results for the constituent ratio were omitted.

Original article: Oncotarget. 2017; 8:72893-72909. https://doi.org/10.18632/oncotarget.20552

